# Functional CRISPR screens in T cells reveal new opportunities for cancer immunotherapies

**DOI:** 10.1186/s12943-024-01987-z

**Published:** 2024-04-05

**Authors:** Minghua Xiang, Huayi Li, Yuanyuan Zhan, Ding Ma, Qinglei Gao, Yong Fang

**Affiliations:** 1 Department of Obstetrics and Gynecology, National Clinical Research Center for Obstetrics and Gynecology, Tongji Hospital, Tongji Medical College, Huazhong University of Science and Technology, Wuhan, China; 2 Key Laboratory of Cancer Invasion and Metastasis (Ministry of Education), Hubei Key Laboratory of Tumor Invasion and Metastasis, Tongji Hospital, Tongji Medical College, Huazhong University of Science and Technology, Wuhan, China; 3 Department of Plastic and Cosmetic Surgery, Tongji Hospital, Tongji Medical College, Huazhong University of Science and Technology, Wuhan, China

**Keywords:** CRISPR screen, T cell, Tumour immunity, Cancer immunotherapy, Adoptive cell therapy, Immune checkpoint inhibitor, T cell exhaustion

## Abstract

T cells are fundamental components in tumour immunity and cancer immunotherapies, which have made immense strides and revolutionized cancer treatment paradigm. However, recent studies delineate the predicament of T cell dysregulation in tumour microenvironment and the compromised efficacy of cancer immunotherapies. CRISPR screens enable unbiased interrogation of gene function in T cells and have revealed functional determinators, genetic regulatory networks, and intercellular interactions in T cell life cycle, thereby providing opportunities to revamp cancer immunotherapies. In this review, we briefly described the central roles of T cells in successful cancer immunotherapies, comprehensively summarised the studies of CRISPR screens in T cells, elaborated resultant master genes that control T cell activation, proliferation, fate determination, effector function, and exhaustion, and highlighted genes (*BATF*, *PRDM1*, and *TOX*) and signalling cascades (JAK-STAT and NF-κB pathways) that extensively engage in multiple branches of T cell responses. In conclusion, this review bridged the gap between discovering element genes to a specific process of T cell activities and apprehending these genes in the global T cell life cycle, deepened the understanding of T cell biology in tumour immunity, and outlined CRISPR screens resources that might facilitate the development and implementation of cancer immunotherapies in the clinic.

## Background

Cancer immunotherapies have drastically changed the paradigm of cancer treatment [1–3]. Ranging from the first IFN-α and IL-2 cytokine therapies to recent adoptive cell therapies (ACTs) and immune checkpoint inhibitors (ICIs), immunotherapies have made tremendous strides in the treatment of multiple cancers [4–6]. Increasing numbers of immunotherapies featured by ICIs and chimeric antigen receptor (CAR)-T cell therapies have received authority approval for cancer treatment [7–11]. T cells are critical effectors in cell-mediated anti-tumour immunity and paramount responders to immunotherapies that improve T cell activation, proliferation, effector function, and persistence [12]. Genetically modified T cells, especially engineered CAR-T cells, display significantly improved anti-tumour efficacy [13]. Despite these prodigious advances, cancer immunotherapies are riddled with challenges of compromised efficacy and resistance induction in multiple cancers, highlighting the priority and urgency to understand the complex and heterogenous T cell phenotypes, decipher the underlying metabolism and regulatory pathways in T cell life cycle against antigens, and reveal pivotal targets that could be leveraged to enhance T cell fitness and maximize the efficacy of cancer immunotherapies.

Genome-wide clustered regularly interspaced short palindromic repeats (CRISPR) gene screens enable unbiased interrogation of gene function and have been applied to discover driver genes and pathways relevant to T cell responses [14, 15]. Previously, functional screens primarily utilized RNA interference (RNAi) to target mRNAs of interest and genetic techniques featured by zinc-finger nucleases (ZFNs) and transcription activator-like effector nucleases (TALENs). RNAi, ZFNs, and TALENs indeed advance the understanding of numerous genes but are restricted by incomplete suppression of target genes, potential off-targets effects, and impaired efficiencies in primary cells. CRISPR systems possess the advantages of increased efficiencies, less cost-consuming gene editing, flexible approaches of both activation and interference, and extensive applications in ex vivo and in vivo in human and mice cells even in primary cells. A series of CRISPR screens in T cells have identified a plethora of novel regulators of T cell development and function and provided additional insights into mechanisms underpinning diverse T cell phenotypes.

In this review, we briefly overviewed the applications of cancer immunotherapies, underscored the significant importance of T cells in successful cancer immunotherapies, and succinctly delineated a roadmap of functional screen methods including RNAi, ZFNs, TALENs, and CRISPR systems. Importantly, we comprehensively reviewed and summarised critical genes and pathways identified by CRISPR screens for T cell activation, proliferation, fate determination, cytotoxicity, and exhaustion in tumour immunity. Since T cell responses to tumours and viral infections have certain similarities characterized by chronic antigen exposure-induced T cell exhaustion, we included several prestigious CRISPR screens in T cells upon viral infection. Finally, we highlighted genes (*BATF*, *PRDM1*, and *TOX*) and signalling cascades (JAK-STAT and NF-κB pathways) that extensively engage in multiple branches of T cell anti-tumour responses, outlined preclinical models to validate the efficacy of manipulating resultant genes for augmented cancer immunotherapies, and depicted the prospects of pharmaceutical inhibition and genetic modification to target these genes in the clinic.

## Main text

### Central roles of T cells in cancer immunotherapies

**Fig. 1:**
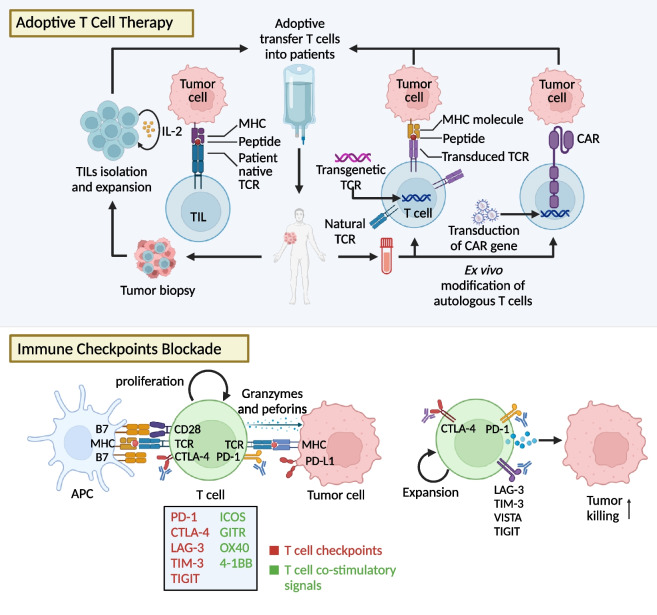
T cell-based cancer immunotherapies. Summarisation of T cell-based immunotherapies for cancer treatment including genetically modified CAR-T cell, TCR-T cell, and TILs therapy, ICIs, and cancer vaccines. 1) TCR-T cell therapy introduces a novel TCR gene into patients-derived T cells, so that engineered TCR molecules can recognize peptides presented on MHC molecules. Of note, TCR-T cell therapy is human leukocyte antigen (HLA)-restricted. 2) CAR-T cell therapy is based on genetic introduction of CARs into autologous T cells. CARs integrate both antigen-binding and costimulatory domains, offering the independence from TCR and HLA restriction. 3) In TILs therapy, tumours are resected from patients, and TILs are isolated and expanded ex vivo. TILs therapy ensures that each antigen from the patients is pre-matched to extracted lymphocytes. 4) ICIs use antibody to inhibit the suppressive signals in T cells featured by PD-1 and CTLA-4, improving T cell priming, proliferation, and cytotoxicity. Novel immune checkpoint combinations, combination of ICIs with targeted therapies, radiation, oncovirus further broaden the application of ICIs. 5) Cancer vaccines activate innate immune system by presenting cancer antigens to immune cells, enabling them to accurately and efficiently recognize tumour cells

T cells play pivotal roles in ACTs including tumour-infiltrating lymphocytes (TILs), CAR-T, and T cell receptor (TCR)-T cell therapies, which involve the extraction of T cells from tumours or peripheral blood mononuclear cells followed by quantitative ex vivo expansion and in some cases, genetic engineering. Manipulated T cells are reinfused to bolster anti-tumour immunity. Besides ACTs, T cell-associated cancer immunotherapies encompass multiple ICIs (Fig. 1).

### TILs therapy

TILs therapy includes the selection and expansion of pre-existing autologous T cells with specificity for tumours to enhance the precision and effectiveness of tumour elimination. After generating a sizable population of activated tumour-specific T cells, revitalized T cells are transferred to patients to mount robust immune responses against tumours [3]. In responders to TILs therapy, the TILs population shows strong recognition of tumour antigens presented by MHC-I and MHC-II molecules. ACT using TILs has shown activity in several cancers. Specifically, in metastatic melanoma, 22% of patients achieved complete tumour regression, while confirmed responses were observed in 23% of patients with metastatic non-small cell lung cancer (NSCLC) [16, 17]. Importantly, improved clinical responses of TILs therapy are associated with the persistence of functional effector T cells (Teff) and a broad spectrum of T cell clonality in vivo after infusion. The prospects of TIL implementation may depend largely on the selection of TIL subpopulations, the differentiation state of TIL, and the durable post-infusion response activity[18, 19]. Tumour-specific CD8^+^ T cells at a subtype of early effector eradicate tumours more effectively than fully effector CD8^+^ T cells [20]. Stem-like T cell clonality with high expression of TCF1 and higher numbers of CD39^−^ CD69^−^ TILs suggests preferable anti-tumour responses of TILs therapy [21]. Consistent with endogenous T cells, transferred T cells enriched with memory features or precursor exhausted T phenotypes can better clonally expand ex vivo and achieve better tumour depletion compared with terminal exhausted T cells (Tex) [22], indicating T cell clonality and phenotypes greatly affect the effectiveness of TILs therapy.

### TCR-T cell therapy

T lymphocytes with heterozygous TCRs can identify HLA peptides on tumour cells and initiate activation signals, therefore activating the immune cells to kill tumour cells. Tumours with low mutation burdens and neoantigens could not be effectively recognized by pre-existing TILs, leading to modest responses to TILs therapy. TCR-T therapy overcomes the limit of surface antigen expression of the target cells, enabling promising cancer cellular immunotherapy. TCR-T cell therapies initiate de novo anti-tumour responses through genetically modifying T cells to target specific tumour-associated antigens (TAAs) and redirecting them to eliminate tumours. Leveraging genetic technologies, T cells can be equipped with tumour-specific TCRs and expand ex vivo to achieve specified functionality. TCR-T cell therapy involves isolation, sequencing, and validation of TCRs that intrinsically target TAAs in an MHC molecule-dependent manner. Engineered TCR-T cells targeting MART1 have achieved partial responses in 2 of 17 patients with MART1^+^ metastatic melanoma, while those targeting NY-ESO-1 yielded objective responses in 11 of 20 patients with NY-ESO-1^+^ melanomas and 11 of 18 patients with NY-ESO-1^+^ synovial cell sarcomas in small-scale clinical trials [23, 24].

Nevertheless, TCR-T cell therapies are confronted with significant challenges. Insufficient activation of T cells causes immune escape and tumour cells can evade immune attack by hiding or altering the antigens that can be recognised by TCR-T cells, deterring the efficacy of TCR-T cell therapies [25, 26]. Besides, side effects such as cytokine release syndrome (CRS) and neurotoxicity are sometimes lethal. In a trial using MAGE-A3-targeted TCR-T cells to treat metastasis melanoma, three of the seven patients developed severe central nerve system damage and died of multifocal necrotizing leukoencephalopathy [27]. TCR-T cell therapy also brings risks of on-target, off-tumour (OTOT) toxicity, which was observed in TCR-T cell therapy targeting melanoma-associated antigens [28]. TCR-T cell therapies targeting carcinoembryonic antigen in metastatic colorectal cancers have mediated tumour regression but induce severe transient colitis, probably because of the normal expression of CEA in healthy colonic mucosa [9]. To circumvent the latent adverse effects, various methods including alanine scanning, X-scan, and positional scanning synthetic combinatorial libraries (PS-SCLs) were developed to evaluate TCR-T affinity and avidity as well as efficiency and safety at the preclinical level [29–31]. Moreover, strategies to enhance clinical responses to TCR-T cell therapy are centred on improving the specificity and avidity of tumour-mutated peptides and identifying tumour neoantigens applicable to a broad spectrum of patients [32–34].

### CAR-T cell therapy

CAR-T cell therapy overcomes the limitation of dependence on MHC molecules and expands the TAAs spectrum so that T cells can recognize and generate anti-tumour responses. Unlike TCRs, CARs can recognize canonical protein-structured antigens and surface targets like carbohydrates and glycolipids because of their MHC-unrestricted recognition mechanism. CARs are engineered fusion proteins that comprise an extracellular single-chain variable fragment of an antibody, a transmembrane spacer structure, and an intracellular signalling domain. The efficacy of CAR-T cell therapy has been well established in patients with relapsed/refractory (R/R) multiple myeloma (RRMM) and B-cell acute lymphoblastic leukaemia. The most impressive clinical responses were observed in the treatment of CD19^+^ R/R B-cell malignancies. CAR-T cell therapies achieved remarkable successes with prolonged remission and demonstrated complete response rates ranging from 71 to 81% with limited adverse effects [9, 28, 32]. CD19-targeted CAR-T cells resulted in high complete response rates ranging from 62 to 86% and exhibited favourable long-term outcomes for patients with B cell lymphoma and chronic lymphocytic leukaemia [33–36]. Phase I/II studies of B-cell maturation antigen (BCMA)-targeted CAR-T cell therapies reported an overall response rate (ORR) of over 70%, complete response rates ranging from 33 to 83%, and varied median progression-free survival in patients with RRMM, indicating prolonged remissions after CAR-T cell therapies [11, 37–39]. CAR-T therapy has demonstrated remarkable efficacy in several haematological malignancies, but it remains challenging in the treatment of solid tumours. Solid tumours are characterised by a hostile tumour microenvironment (TME) filled by immunosuppressive cytokines featured by TGF-β, IL-4, and IL-10, strikingly hindering T cell infiltration and weakening the persistence and effector function of T cells [40–42]. Enhancing the potency of CAR-T cells for solid tumour targeting could be enabled by inducing resistance against exhaustion, promoting the formation of memory cells, and preselecting naïve/stem memory T cells [43, 44]. In vivo mouse models discovered that overexpressing canonical AP-1 factor c-Jun in CAR-T cells enhanced CAR-T cell expansion, rendered resistance to T cell exhaustion, and augmented anti-tumour efficacy [45]. In addition, it was proposed that the deletion of *Cbl-b* in CAR-T cells could alleviate exhaustion and enhance the effectiveness of CAR-T cell therapy in solid tumours [46].

It is noted that CAR-T cell therapy is still riddled with challenges that require solutions. For instance, cytokine release syndrome (CRS) and neurotoxicity (referred to as ICANS, immune effector cell–associated neurotoxicity syndrome) are probably caused by the combined impact of CD28 and 4-1BB co-stimulatory domains, which have been identified in large-cohort clinical trials [47]. Another significant concern in CAR-T cell therapy is the target antigens expressed both on the surface of cancer cells and normal cells. Even when the expression levels of target antigens are low in normal cells, the OTOT toxicities can sometimes be lethal [48, 49]. In a phase 1 dose-escalation study of patients with solid tumours, two patients experienced severe pulmonary toxicity after the infusion of CAR-T cells targeting mesothelin (MSLN), ultimately leading to fatalities[50].

Encouragingly, mounting efforts have been made to mitigate OTOT toxicity and to enhance potency and reduce exhaustion in CAR-T cells [51, 52]. Novel design of CAR-T cells significantly contributes to the improvement of their anti-tumour efficacy. Enabled by CRISPR genome editing, CD19-specific CAR is delivered to the T-cell receptor α constant (TRAC) locus, which results in enhanced T cell activity and slows the development of T cell exhaustion [53]. Redesigned CAR-T cells targeting tumour-specific driver gene mutations neoepitope, human leukocyte antigen with the A2 serotype (HLA-A2^+^), effectively alleviates the OTOT toxicity in therapies of acute myeloid leukaemia (AML) [54]. New types of CAR-T cells with its CAR construct of BCR light chain neoepitope composed of a characteristic point mutation (IGLV3-21(R110)) are able to selectively target poor-risk subset of chronic lymphocytic leukaemia (CLL), exert epitope-selective cytolysis effects, and effectively protect normal human B cells [55]. To overcome allo-recognition, a novel platform coupling CAR expression with CRISPR/Cas9 gene editing system has been established to enable pre-manufactured, non-HLA-matched "universal" CAR-T cell therapy [56]. Moreover, efforts to maintain persistent CAR-T responses indicate that disrupting methylcytosine dioxygenase TET2 could alter T cell differentiation towards a central memory state and promote the therapeutic efficacy of CD19-targeted CAR-T cells [57].

### ICIs

ICIs exert effects by braking inhibitory signals that impede T cell activation, thereby reinvigorating anti-tumour T cell responses. CTLA-4, the first discovered immune checkpoint, was identified as a negative influencer of antigen-presenting cells (APCs)-induced T cell responses. Since ipilimumab was approved for the treatment of unresectable or metastatic melanoma, ICIs have provided effective and durable responses in various cancers including renal cell carcinoma (RCC), NSCLC, colorectal cancer, and cutaneous squamous cell carcinoma. While several immune checkpoints including LAG-3, TIM-3, and TIGIT are still in preclinical stages, targeting these checkpoints alone or in combination have shown remarkable efficacy in tumour eradication. These checkpoints have different action of mechanisms to brake T cell activation and heterogenous expression in T cell subtypes, which have been systematically summarised in excellent reviews[58, 59]. Combining therapies targeting checkpoints could further increase clinical responses. Blocking TIM-3 combined with PD-1 blockade overcomes the resistance to PD-1 blockade in head and neck cancer [60], and nivolumab plus ipilimumab reached enhanced anti-tumour responses in B16 melanoma, RCC, NSCLC, mesothelioma, gastro-oesophageal cancer, and hepatocellular carcinoma (HCC) [8, 61–65]. Moreover, ICIs have synergistic effects with surgery, radiotherapy, chemotherapy, targeted therapies [66–69], and ACTs including CAR-T cell therapy and TILs therapy [70–73]. ICIs display elevated efficacy in immunologically “hot” tumours with large amounts of pre-existing CD8^+^ TILs [74]. The effectiveness of ICIs is also associated with the functional status of T cells, with precursor exhausted T cells in the TME often considered as a key factor contributing to ICI efficacy[75]. Meanwhile, different T cell status (activation, memory, exhaustion and anergy) presents various metabolism patterns, and investigations concerning how T cell metabolism can regulate the efficacy of ICIs in solid tumours are of mounting interest for researchers[76].

### Challenges of cancer immunotherapies

Though immunotherapies have shown encouraging clinical responses in multiple cancers, there are still challenges and areas of uncertainty regarding the comprehension of infiltration of T cells into tumours, avidity of peptide-HLA-TCR interactions within the spectrum of tumour antigens, and selection of tumour-reactive TCRs by genetic profiling [77]. Anti-tumour capabilities of T cells are characterized by TCRs activation, recognition of TAAs, and HLA molecules presented by APCs, especially dendritic cells (DCs). The direct, sufficient, and effective encounters with antigens guarantee successful anti-tumour T cell responses. However, T cells often fail to get primed in tumour surroundings due to 1) tumour antigen escape and decreased expression of TAAs, 2) defection of tumour antigen presentation and MHC loss, and 3) suppressive interactions between APCs and T cells. Besides, T cells inevitably develop exhaustion phenotypes when exposed to chronic antigen exposure within TME. Tex reduces the secretion of effector cytokines featured by IFN-γ and granzyme B, upregulates exhaustion markers including PD-1 and TIM-3, and undergoes extensive transcriptional changes compared to Tex or memory T cells (Tmem). T cell immunity is also influenced by inhibitory cytokines and signalling factors within TME, which hinder T cell proliferation and cytotoxicity, reprogram T cell clonotypes, and engender dysfunctional regulatory T cells (Tregs) [75, 78, 79]. To enhance anti-tumour T cell immunity and potentiate cancer immunotherapies, it is critical to deepen the understandings of T cell biology, uncover master genes and underlying mechanisms of T cell responses in tumours, and decode the cellular and molecular interactions shaping T cell responsiveness.

### CRISPR screens expand the crucial genetic regulators of T cell functions

**Fig. 2:**
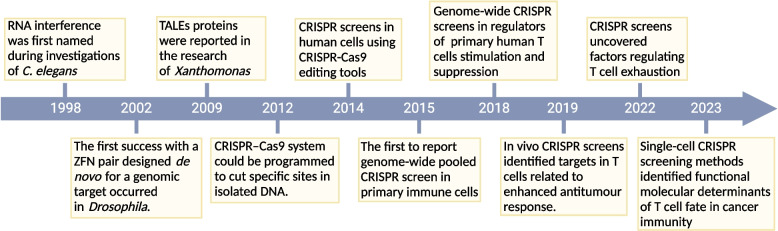
Timeline of major milestones in researches of genetic engineering and T cell-associated functional screens. Researches of genetic engineering and T cell-associated functional screens are depicted. Preceding the advent of CRISPR-Cas9 system, gene editing relied on RNAi, ZFNs, and TALENs, facilitating targeted mutations in cells and organisms. The introduction of CRISPR-Cas9 systems ushered in more convenient approaches for manipulating gene expression. The emergence of genome-wide CRISPR screens and studies dedicated to T cell functional screening expanded our comprehension of the intricate yet pivotal roles of T cells in cancer immunity

The roadmap of functional screens in T cells is hallmarked by several milestones including the applications of RNAi, ZFNs, TALENs, and CRISPR systems (Fig. 2).

RNAi is an endogenous cellular process initially discovered in *Caenorhabditis elegans* and remains conserved in most eukaryotic species. High-throughput gene silencing can be enabled by introducing RNAi reagents targeting endogenous mRNA transcripts [80–82], making RNAi an effective tool for genome-scale, high-throughput analysis of gene function. RNAi has made major progresses in elucidating the regulatory networks and intrinsic metabolisms of T cells in viral infections, autoimmune disorders, and tumour immunity [83–87]. For example, genome-wide RNAi screening identified eukaryotic translation eIF4A2 as a key factor governing HIV replication in human T cells, providing a promising clinical target of HIV therapy[88, 89]. RNAi screens targeting signal transduction revealed that NF-κB-related kinase PP4R1 negatively regulates T cell stimulation and inhibits developmental NF-κB signal transduction [85]. In vivo studies using RNAi screens in mice indicated bromodomain-containing protein 4 (BRD4) as therapeutic targets in acute myeloid leukaemia (AML), and positive transcription elongation factor (P-TEFb) component Cyclin T1 (Ccnt1) and catalytic partner Cdk9 as regulators of antiviral CD4^+^ and CD8^+^ T cell differentiation [87, 90]. Unlike RNAi-based screens, ZFNs and TALENs rely on protein-DNA interactions and introduce double-stranded breaks (DSBs) to enable the deletion and addition of DNA sequences for genome editing. ZFNs and TALENs have escalated knock-out specificity but require custom design of targets, lacking the ability of screening in bulks of target genes. Moreover, targeting genome sequences using TALENs requires DNA–protein interactions and adds the difficulty of multi-targeting in cell pools [91–96].

CRISPRs are DNA sequences discovered in prokaryotic organisms. Upon exposure to viral infection, foreign genetic elements are cleaved by CRISPR-associated protein (Cas) and integrated into CRISPR locus, forming a specific CRISPR array and providing bacteria with memory of prior infections. CRISPR systems have been adapted to a more simple-structured and high-efficiency genome editing tool in eukaryotic organisms. CRISPR systems are composed of a single-guide RNA (sgRNA) sequence synthesized in laboratories based on target gene sequences and Cas9 nuclease from Streptococcus pyogenes, which is simultaneously delivered using a lentiviral system. Once Cas9 protein binds to target locus and cleaves the complementary gene array, DSBs occur and DNA repair mechanisms including non-homologous end joining (NHEJ) are activated. NHEJ DNA repair machinery does not require a template and the reconnection is randomized, potentially leading to the dysfunction of target genes.

**Fig. 3:**
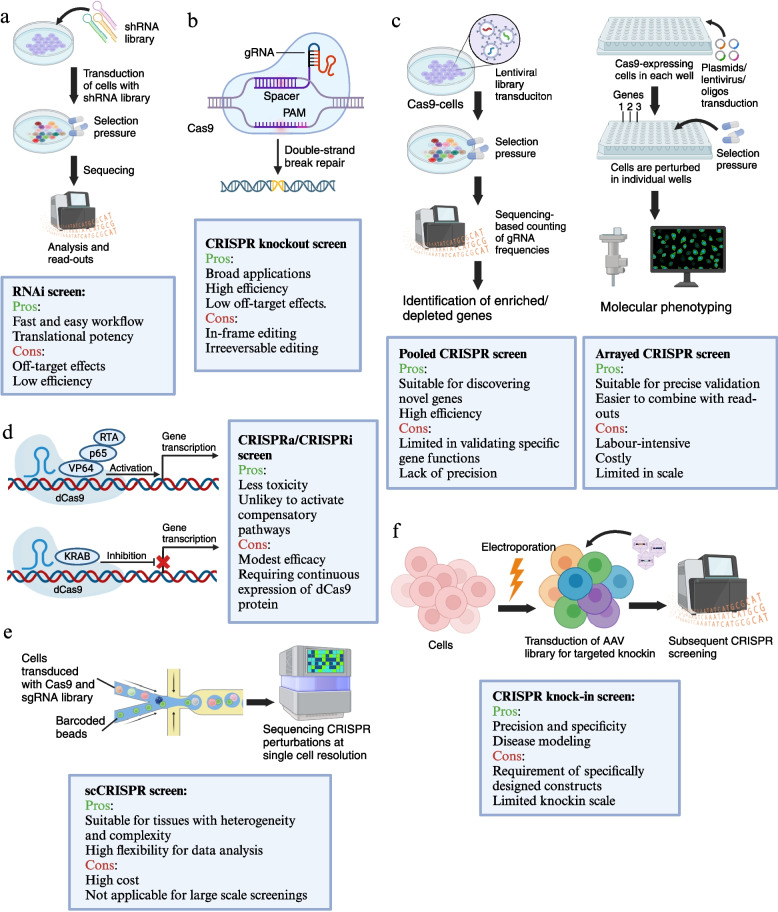
Different CRISPR screening methods are described with their applications, pros and cons. **(a)** RNAi screen. In RNAi screens, RNAi reagents were introduced into the cells to target the endogenous mRNA transcripts. By combining RNAi tools with analysis such as signal transduction, cell viability, and responses to infections, RNAi screening enables identification of new genes, and the information of gene function in a wide variety of biological processes. **(b)** CRISPR knockout screen. Directed by a guide RNA (gRNA), Cas9 nucleases introduce double-strand breaks (DSB) into the target site; subsequent DNA repair results in compromised gene function. **(c)** Pooled/arrayed CRISPR screen. Pooled CRISPR screens introduce perturbations in bulk, genetically encoding them, and commonly employing gRNA sequencing for readout. Arrayed CRISPR screens involve the separate introduction of distinct perturbations. Since each reaction compartment undergoes a defined perturbation, the read-out does not necessarily require gRNA sequencing. **(d)** CRISPRa/CRISPRi screen. CRISPR activation (CRISPRa) screen employs dCas9 coupled with transcriptional activators, such as the VP64 domain, resulting in the stimulation of genes in target site. CRISPR interference (CRISPRi) screen uses dCas9, fused with transcriptional repressors like Krüppel-associated box (KRAB). This fusion results in the repression of genes in proximity to the gRNA target site. **(e)** scCRISPR screen. scCRISPR (single-cell CRISPR) screens combine pooled CRISPR perturbations with scRNA-seq, offering opportunities to investigate genome regulatory networks by interrogation of different perturbations with the transcriptome profiles at single-cell resolution. **(f)** CRISPR knock-in screen. CRISPR-based knock-in screens mediate simultaneous gene editing and precise transgene knock-in, producing cell pools with targeted stable gene editing

CRISPR screenings have gained widespread popularity in the field of tumour immunotherapy, especially in the discovery of crucial targets associated with T cells. CRISPR screenings offer powerful methodology for understanding genes, pathways, and mechanisms implicated in a specific phenotype or biological process. The maturation and commercialization of sgRNA libraries and abundant CRISPR-based perturbation methods have enabled large-scale, high-content, and high-efficiency genome functional screenings. Current screening strategies using CRISPR technologies are depicted in Fig. 3.

In a typical pooled CRISPR screening, thousands of sgRNAs are delivered to pools of cells, with each cell receiving a sgRNA sequence. Next, CRISPR-edited cells undergo rigorous examination and are subjected to selective pressures. Subsequently, the abundance of guide RNAs (gRNAs) is quantified within the cell pool, typically by high-throughput genome sequencing. In read-outs, pooled CRISPR screens require sequencing-based counting of gRNA frequencies to evaluate the cells with different perturbations. The depletion of specific gRNAs reveals genes whose disturbance increases susceptibility to the challenge. Conversely, their enrichment represents genes whose disruption brings a selective advantage [97–99]. In arrayed CRISPR screens, individual perturbations are introduced, with each target gene occupying a separate compartment. Since a specific perturbation is applied to each reaction compartment, the read-out does not necessitate gRNA sequencing. Considering the scale and scope, arrayed screens are primarily employed for validation and subsequent investigations of specific molecules and signalling pathways, while pooled CRISPR screens are suitable for discovery and high-content selection in large gene sets [100, 101].

Currently, most CRISPR screens utilize CRISPR knockout (CRISPRko) technology to enable precise targeting of gene sequences for accurate knockout. However, CRISPRko is irreversible and may cause significant toxicity to cells. Therefore, alternative technologies known as CRISPR activation (CRISPRa) and CRISPR interference (CRISPRi) have emerged. These approaches leverage dCas9 nuclease, a deactivated Cas9 protein that cannot cut DNA, along with the recruitment of activation/suppression transcriptional proteins, to achieve the activation or inhibition of gene sequences. Specifically, CRISPRi uses dCas9 and transcriptional repressors such as the Kruppel-associated box (KRAB) domain to inhibit target genes, while CRISPRa enables combination of dCas9 and transcriptional activators such as VP64 to stimulate the transcription at targeted locus [102–104].

Recently, novel CRISPR-based perturbations are put forward, such as single-cell CRISPR (scCRISPR) screens and CRISPR knock-in screens. High-throughput CRISPR knock-in screens mediate simultaneous gene editing and precise transgene knock-in, producing cell pools with targeted stable gene editing [105, 106]. scCRISPR screens combine pooled CRISPR perturbations with single-cell RNA sequencing (scRNA-seq), offering opportunities to investigate genome regulatory networks by interrogation of different perturbations with the transcriptome profiles at single-cell resolution [105, 107]. The various CRISPR perturbations strategies provide us with valuable research platforms for studying gene functions and the intricate interactions among cells, cytokines, and the microenvironment. Since CRISPR-based perturbations have been delicately reviewed elsewhere [99, 106, 108–110], herein we focus on how CRISPR screens depict T cell function and shed light on the future roadmap of T cell-related cancer immunotherapies.

### CRISPR screening in T cells uncover critical genes in T cell life cycle

**Fig. 4:**
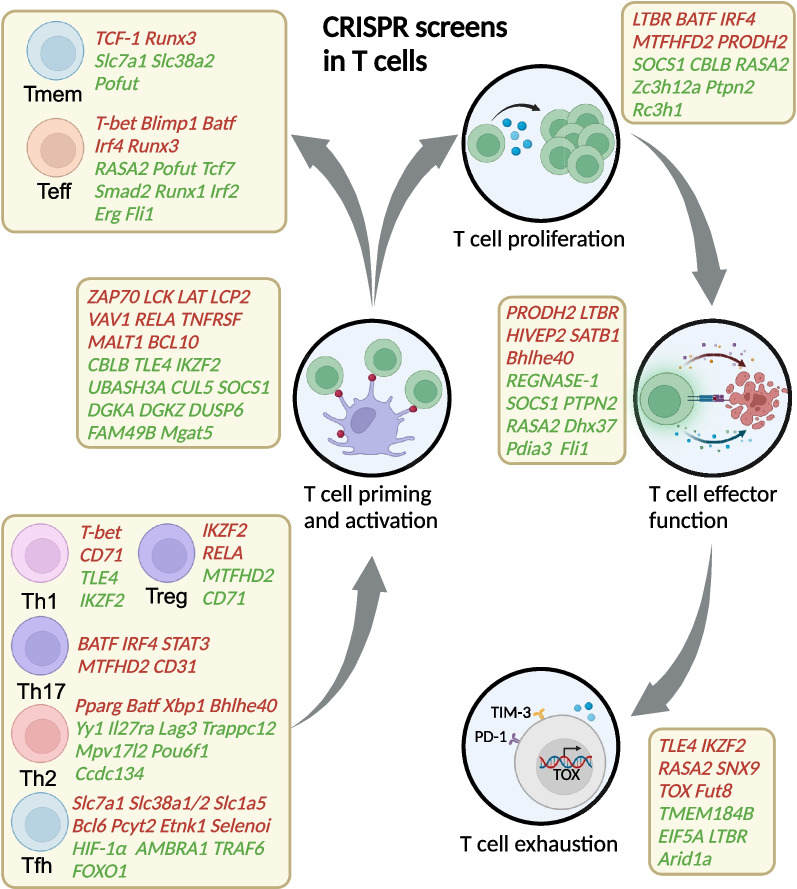
Summarisation of CRISPR screen results according to stages of T cell life cycle. CRISPR screens have successfully identified crucial regulatory factors and networks central to T cell immunity in the context of cancer treatment. High-throughput CRISPR screens were conducted to identify genes that govern various aspects of T cell function including activation, proliferation, differentiation, effector function, and exhaustion. Positive regulators are indicated in red, whereas negative regulators are represented in green

T cell anti-tumour activity is generated by unique TCRs, with the recognition of tumour antigens presented by the HLA complex. Following successful T cell stimulation, these cells travel through the bloodstream and infiltrate tumour-bearing sites, where T cells generate robust responses characterized by phenotype differentiation, cytokine production, and memory induction [111]. Within tumour sites, chronic antigen exposure and immunosuppressive TME lead to T cell exhaustion and compromised T cell responses [76, 112]. CRISPR screens have been extensively applied in T cells to identify crucial regulators of T cell activation, proliferation, differentiation, cytotoxicity, and exhaustion (Fig. 4 and Table 1), providing promising targets for cancer immunotherapies.

**Table 1:** CRISPR screens in T cells to identify genes for cancer immunotherapy

Conditions	Cells	Libraries	Methods	Purpose	Hit genes	References
ex vivo	IL13Rα2-targeted CAR T cells	Addgene #73179	CRISPR loss of function screens	CAR-T effector activity, cytotoxic potency	*TLE4 IKZF2*	Wang, D., et al. (2021)[113]
ex vivo	Primary human T cells	Addgene #73178	CRISPR loss of function screens	T cell proliferation	*SOCS1 TCEB2 RASA2 CBLB*	Shifrut, E., et al. (2018)[114]
ex vivo	Primary human T cells	lentiSAM v2	Coupled CRISPR activation and CRISPR interference screen	Gene networks controlling IL-2 and IFN-g production	*VAV1 CD28 LCP2 LAT MALT1 BCL10 TRAF6*	Schmidt, R., et al. (2022)[115]
ex vivo	Primary mouse CD8^+^ T cells	mm10dgLib	dead-guide RNA (dgRNA)-based CRISPR activation screen	CAR-T killing and in vivo efficacy in cancers	*PRODH2*	Ye, L., et al. (2022)[116]
in vivo	P14-transgenic Cas9-expressing CD45.1^+^ LSK cells (mouse)	A library of 110 sgRNAs targeting 21 genes relevant to T cell biology and 50 non-targeting control sgRNAs	CHIME: CHimeric IMmune Editing,	CD8^+^ T cell responses to LCMV Clone 13 infection	*Ptpn2*	LaFleur, M. W., et al. (2019)[96]
in vivo and ex vivo	Primary mouse CD8^+^ T cells	The genome-scale mouse T cell CRISPR library (MKO)	CRISPR loss of function screens	T cell activation, tumour infiltration and degranulation	*Dhx37*	Dong, M. B., et al. (2019)[117]
in vivo	Primary mouse CD8^+^ T cells	sgRNA library targeting 1658 genes, with 6628 sgRNAs and 1000 nontargeting controls	AAV–SB-CRISPR screens	T cells effector functions, and potent of killing tumour cells	*Lag3 Mgat5 Pdia3 Emp1*	Ye, L., et al. (2019)[118]
ex vivo	Primary human CD4^+^ T cells	Human genome-wide sgRNA library targeting 18,360 protein-coding genes	Guide Swap pooled CRISPR enrichment and depletion screens	Validation of guide swap in depletion and enrichment screens in CD4^ +^ T cells	*MDM2 WEE1 EEF2 TOP2A*	Ting, P. Y., et al. (2018)[119]
ex vivo	68–41 murine T cell line	Lentiviral gRNA library (87,898 gRNAs/19,150 genes)	CRISPR loss of function screens	T cell activation	*Fut8*	Okada, M., et al. (2017)[120]
ex vivo	Primary mouse T helper cells	Addgene #67988	CRISPR loss of function screens	T helper cell activation and differentiation	*PPARG*	Henriksson, J., et al. (2019) [121]
in vivo	Mouse CD8^+^ T cells	Pooled gRNA library of 3017 metabolism-associated genes	CRISPR loss of function screens	CD8^+^ T cell fate decision	*Pofut1*	Huang, H., et al. (2021)[122]
in vivo	Mouse CD8^+^ T cells	Two lentiviral sub-libraries of single-guide sgRNAs targeting 3017 metabolic enzymes, small molecule transporters and metabolism-related transcriptional regulators	CRISPR loss of function screens	T cell longevity and antitumour response	*REGNASE-1*	Wei, J., et al. (2019)[123]
in vivo	Antigen-experienced mouse CD4^+^ T cells	A genome-wide knockout (GWKO) sgRNA lentiviral library (18,400 genes, 90,000 sgRNAs)	CRISPR loss of function screens	The expansion of activated CD4^+^ T cells	*SOCS1*	Sutra Del Galy, A., et al. (2021)[97]
in vivo	Primary mouse CD8^+^ T cells	Retroviral sgRNA library targeting 271 transcription factors	CRISPR loss of function screens (OpTICS)	T cell effector activity	*Fli1*	Chen, Z., et al. (2021)[124]
in vivo and ex vivo	Chronic stimulated CD8^+^ T cells	Addgene #104861	CRISPR loss of function screens	T cell exhaustion	*Arid1a*	Belk, J. A., et al. (2022)[125]
ex vivo	Jurkat T cells	sgRNA library consists of over 250,000 total sgRNAs targeting every unique Refseq annotated (hg19) protein coding isoform with up to 12 sgRNAs, plus 7700 NTCs	CRISPR loss of function screens	T cell activation	*FAM49B*	Shang, W., et al. (2018)[126]
ex vivo	Human CD4^+^ and CD8^+^ T cells	Lentiviral library of human ORFs containing nearly 12,000 full-length genes	CRISPR gain of function screens	T cell proliferation	*LTBR*	Legut, M., et al. (2022)[127]
in vivo	Mouse OT-1 CD8^+^ T cells	scCRISPR library containing 180 TFs with 720 sgRNAs and 80 NTC sgRNAs	CRISPR loss of function screens	CD8^+^ cytotoxic T cells differentiation and Tex cell reinvigoration	*IKZF1 ETS1*	Zhou, P., et al. (2023)[128]
ex vivo	Primary human CD8^+^ T cells	Published highly optimized library that encodes gRNAs and Cas9 (Wang et al. 2017)	CRISPR loss of function screens	T cell exhaustion	*SNX9*	Trefny, M. P., et al. (2023)[129]
ex vivo	Primary mouse CD8^+^ T cells	sgRNA library from Benchling or Brie, adding up to a total of 2298 sgRNAs	CRISPR loss of function screens	T cell adhesion, migration, homeostasis	*RASA3*	Johansen, K. H., et al. (2022)[130]
ex vivo	Primary human T cells	Brunello sgRNA library	CRISPR loss of function screens (SLICE)	T cell dysfunction	*RASA2*	Carnevale, J., et al. (2022)[131]
ex vivo	Primary mouse Treg cells	A library of 489 nuclear factors on the basis of optimized sgRNA sequences from the Brie library	CRISPR loss of function screens	Treg instability	*Usp22 Rnf20*	Cortez, J. T., et al. (2020)[98]
in vivo	Primary mouse CD4^+^ T cells	Addgene, PooledLibrary #73632	CRISPR loss of function screens	T cell differentiation and function	*MTHFD2*	Sugiura, A., et al. (2022)[132]
ex vivo	Primary mouse Treg cells	LentiCRISPRv2-Brie	CRISPR loss of function screens	Treg cell function and anti-tumour immunity	*Brd9-*containing *ncBAF* complex	Loo, C. S., et al. (2020)[133]
ex vivo	Primary human Treg cells	Cas9 RNPs pooled containing 40 selected candidate TFs of Treg cell identity	CRISPR loss of function screens	Treg cell function	*HIVEP2 SATB1*	Schumann, K., et al. (2020)[134]
in vivo	Primary mouse CD4^+^ T cells	sgRNA metabolic library	CRISPR loss of function screens	Tfh cell differentiation	*ETNK1 PCYT2 SELENOI*	Fu, G., et al. (2021)[135]
ex vivo	Primary mouse CD4^+^ T cells	Lentiviral Brie sgRNA library (78,633 sgRNAs targeting 19,674 genes)	CRISPR loss of function screens	T cell priming and Treg suppressive function	*SEC31A CCDC101*	Long, L., et al. (2021)[136]
in vivo	Primary mouse CD4^+^ T cells	~ 400 SgRNAs from Brie library targeting ~ 80 genes	CRISPR loss of function screens	Tfh cell development, Tfh versus Th1 decisions	*HIF-1α*	Huang, B., et al. (2022)[137]
ex vivo	Human T cells	sgRNA library targeting 829 genes	CRISPR loss of function screens	T cell exhaustion	*IKZF1*	Gayathri, B., et al. (2022)[138]
in vivo and ex vivo	Primary mouse CD8^+^ T cells	sgRNA library with 78,633 sgRNAs targeting 19,674 genes	CRISPR loss of function screens	CD8^+^ T cell expansion and anti-tumour immunity	*Roquin*	Zhao, H., et al. (2021)[139]

*AAV* adeno-associated virus, Arid1a AT-rich interactive domain-containing protein 1A, *Dhx37* DEAH-box helicase 37, Fli1, friend leukaemia integration 1 transcription factor, *LCMV* lymphocytic choriomeningitis virus, *LTBR* lymphotoxin beta receptor, *HIF-1α* Hypoxia-inducible factor 1-alpha, *RASA2* RAS P21 Protein Activator 2, *RASA3* RAS P21 Protein Activator 3, *Pofut1* Protein O-Fucosyltransferase 1, *PPARG* Peroxisome proliferator-activated receptor gamma, *Fut8* Fucosyltransferase 8, *SOCS1* Suppressor of cytokine signalling 1, *FAM49B* Family with Sequence Similarity 49, Member B; SNX9 Sorting Nexin 9, *IKZF1* IKAROS family zinc finger protein 1, *ETS1* ETS Proto-Oncogene 1, *Ptpn2* Protein Tyrosine Phosphatase Non-Receptor Type 2, *PRODH2* Proline Dehydrogenase 2, *MALT1* Mucosa-Associated Lymphoid Tissue Lymphoma Translocation Protein 1, *BCL10* B-cell lymphoma/leukaemia 10, *TRAF6* TNF Receptor Associated Factor 6, *VAV1* Vav Guanine Nucleotide Exchange Factor 1, *LCP2* Lymphocyte Cytosolic Protein 2, *LAT* Linker For Activation Of T Cells, *CBLB* Cbl Proto-Oncogene B, *TLE4* TLE Family Member 4, *IKZF2* IKAROS Family Zinc Finger 2, *Usp22* Ubiquitin Specific Peptidase 22, *Rnf20* Ring Finger Protein 20, *MTHFD2* Methylenetetrahydrofolate Dehydrogenase 2, *HIVEP2* Human Immunodeficiency Virus Type I Enhancer-Binding Protein 2,*SATB1* Special AT-Rich Sequence Binding Protein 1, *ETNK1*, Ethanolamine Kinase 1, *PCYT2* Phosphate Cytidylyltransferase 2, *SELENOI* Selenoprotein I, *SEC31A* SEC31 Homolog A, *CCDC101 Roquin (RC3H1)* Ring finger and CCCH-type domains 1

### T cell activation

**Fig. 5:**
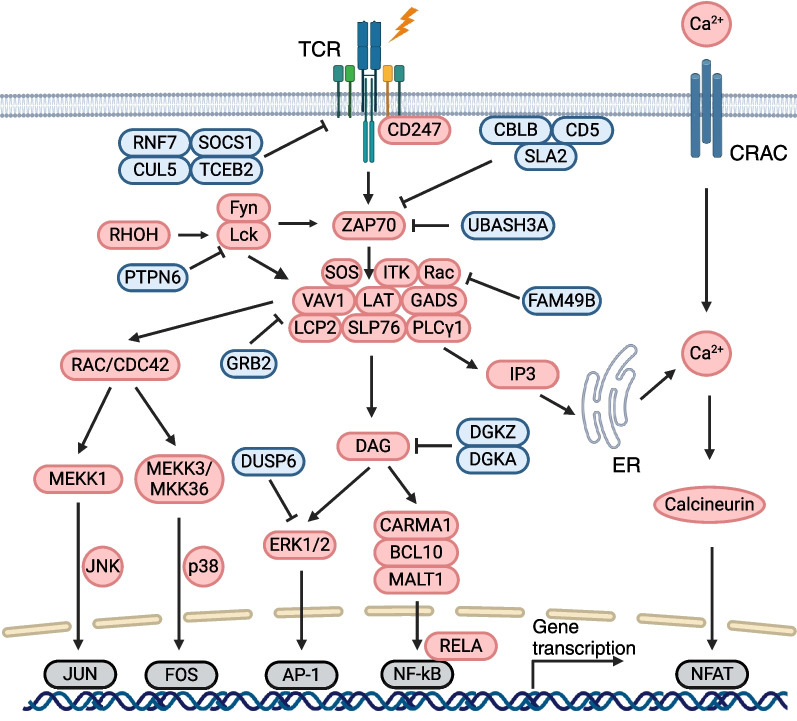
Overview of the major TCR signalling pathways. The major signalling components and transcription factors involved in the transduction of TCR signals are shown in the figure. The red, blue, grey boxes represent stimulatory, inhibitory, and transcription factors, respectively. AP-1, activator protein 1; BCL10, B-cell lymphoma/leukaemia 10; CARMA1, CARD-containing MAGUK protein 1; CRAC, calcium release activated channel; CUL5, cullin-5; DAG, diacylglycerol; DGKA, diacylglycerol kinase alpha; DGKZ, diacylglycerol kinase zeta; DUSP6, dual specificity phosphatase 6; ERK, extracellular signal-regulated kinase 1; FAM49B, family with sequence similarity 49 member B; Fyn, FYN proto-oncogene; GRB2, growth factor receptor-bound protein 2; LAT, linker for activation of T cells; MEKK1, mitogen-activated protein kinase kinase kinase 1; IP3, inositol-1,4,5-trisphosphate; ITK, IL2 inducible T cell kinase; NFAT, nuclear factor of activated T cells; NF-κB, nuclear factor kappa-light-chain-enhancer of activated B cells; PLC-γ1, phospholipase C-gamma 1; PTPN6, protein tyrosine phosphatase non-receptor type 6; RNF7, ring finger protein 7; RHOH, Ras homolog family member H; SOCS1, suppressor of cytokine signalling 1; SLA2, Src like adaptor 2; TCEB2, elongin B; TCR, T cell receptor; UBASH3A, ubiquitin associated and SH3 domain containing A; VAV1, vav guanine nucleotide exchange factor 1; Zap70 ζ-chain associated protein kinase of 70 kDa

T cell activation is a complex process initiated by the interactions between TCRs and cognate antigens presented by APCs, followed by downstream canonical and non-canonical signal transduction cascades (Fig. 5). Cytokines and pathways related to TCR signalling and T cell activation have been identified and characterized using genetic and biomedical approaches. Recently, an in vitro high-throughput CRISPR platform has reclassified key complexes critical for downstream TCR signalling [114]. The ζ-chain associated protein ZAP70 holds the central position in TCR signalling, transmitting the activation signal from CD3 and immunoreceptor tyrosine-based activation motifs (ITAMs) within the TCR-CD3 complex. CRISPR screening identified Src kinases, Lck and Fyn, as well as Rhoh, a GTPase expressed in the hematopoietic system, can phosphorylate ZAP70, leading to T cell downstream signalling. Cbl Proto-Oncogene B (CBLB) and CD5 can degrade TCR complex, directly inhibiting TCR signal transduction. The adaptor protein Src-like adaptor 2 (SLA2) negatively regulates TCR signalling by linking ZAP70 with CBLB, enabling ubiquitin degradation of TCR complex. Phosphorylation of membrane adaptor LAT further activates ZAP70, and fully activated ZAP70 forms an effective signalling complex, which was facilitated by the recruitment of numerous positive regulators (VAV1, GADS, PLCγ1, and SOS). Followed by the activation of LAT-recruited effector molecules, activation of T cells was triggered in cytosol and nucleus mainly through pathways of calcium-calcineurin, MAPK, and NF-κB. Using CRISPR-Cas9 screening methods, critical regulators of TCR downstream signalling pathways were discovered including Calcineurin, *PLCG1*, *PLCG2*, *PRKCB*, *PRKD2*, and *NFATC2* that regulate calcium-calcineurin, *ERK1* and *DUSP6* that inhibit ERK signalling, and negative regulators of DAG signalling, *DGKA* and *DGKZ* [114]. These factors not only regulate TCR activation transduction, but also regulate T cell proliferation through modulating cytokine productions such as IL-2 and IFN-γ, which was examined in genome-wide CRISPRa and CRISPRi screens targeting cytokine production mechanisms in primary human T cells. This reciprocal screening identified that TCEB2, RNF7, CUL5, and SOCS1 suppress JAK/STAT signalling. While UBASH3A, TNFAIP3, and TNIP1 inhibit NF-κB signals, RELA, IL1R1, TRAF3IP2, TNFRSF1A, and TNFRSF1B activate NF-κB signalling pathway, which is a critical pathway for T cell survival after stimulation [97, 114, 115]. FAM49B is a previously unidentified regulator of actin cytoskeleton enriched in lymphoid organs and associates with the pathogenesis of multiple sclerosis. A genome-wide CRISPR screen focusing on key regulators of TCR stimulation discovered that FAM49B deletion in T cells reinvigorates GTPase Rac, enhances cytoskeletal reprogramming, and upregulates CD69, a well-established marker of early T cell activation [126]. Collectively, these studies unveiled promising targets capable of triggering TCR stimulation, which helped T cell signal transduction and facilitated T cell activity.

### T cell proliferation

T cells proliferate and diversify their functional capabilities through differentiation into heterogenous clonotypes of T cells. Genome-scale CRISPR screens within T cells discovered supportive and detrimental factors for T cell proliferation. Leveraging a library of over 12,000 barcoded human open reading frames (ORFs), CRISPRa and CRISPRi screens in primary human T cells identified that forced expression of LTBR can enhance the proliferation of both CD4^+^ and CD8^+^ T cells by inducing NF-κB signalling pathway. The overexpression of LTBR in CD4^+^ T cells can augment IL-2 secretion, thereby playing a synthetic role in promoting T cell proliferation [127]. In antigen-experienced CD4^+^ T cells, a genome-wide CRISPR screen revealed that SOCS1 functioned as a checkpoint to inhibit the proliferation of human and murine CD4^+^ T cells, while exerting little impact on CD8^+^ T cell proliferation [95, 97]. In an in vivo pooled CRISPR–Cas9 mutagenesis screen, REGNASE-1 deletion in CD8^+^ T cells could reprogram their proliferative characteristics within TME. In addition, SOCS1 and PTPN2 were inhibited in *REGNASE-1*-null CD8^+^ T cells, resulting in robust proliferation of effective CD8^+^ T cells in tumours [123]. Furthermore, the integration of in vivo primary T cell gain-of-function CRISPR screens and analysis of differentially expressed genes identified mitochondrial metabolism determinant factors, *MTHFD2* and *PRODH2*, as potential targets that enhance T cell proliferation in ACTs. Specifically, MTHFD2 plays a crucial role in one-carbon metabolism, and its deficiency can impair CD4^+^ T cell proliferation due to deprived purine pools and diminished nutrient sensor mTORC1 signalling. The overexpression of PRODH2, an enzyme involved in proline metabolism, enhances CAR-T cell proliferation through the reprogramming of proline metabolism and the promotion of mitochondrial proliferation [116, 132, 140]. Combined in vivo and in vitro genome-wide CRISPR screens of genes related to CD8^+^ T cell fitness identified Roquin-IRF4 axis as a pivotal component in T cell expansion and anti-tumour immunity. CD8^+^ T cell expansion in vivo is repressed by Roquin, an RNA-binding protein that interacts with Irf4, whereas ablation of roquin drastically boosts CD8^+^ T cell proliferation[139].

### T cell differentiation

Several canonical transcription factors (TFs) have been well appreciated to determine T cell fates including T-bet for type 1 T helper (Th1) cells, GATA3 for type 2 T helper (Th2) cells, and Foxp3 for Tregs. In vivo and in vitro CRISPR screens reaffirmed the significance of classical TFs, and uncovered novel targets in T cell differentiation. CRISPR screens enable stable and flexible modification of T cells, thereby introducing higher specificity and a broader range for identifying governing factors in T cell differentiation. In a CRISPR screen of iron handling genes in T cells, CD71 was identified as a critical receptor that enhances intracellular iron and mTORC1 signalling, promoting Th1 cells reproduction [141]. Moreover, CRISPR screens in CAR-T cells discover that knock-out of *TLE4* or *IKZF2* in CAR-T cells can upregulate Th1 differentiation regulators, BCAT and EGR1, thus polarizing differentiation pathway towards Th1 cells [113]. Genome-wide retroviral CRISPR screens also dissected the differentiation networks of Th2 cells, which play active roles in combating parasites and triggering allergies. Utilizing fluorescent reporters driven by the promoter of genes (*Gata3*, *Il4*, *Il13*, *Xbp1*, and *Irf4*) and antibodies targeting *IRF4*, *XBP1*, *GATA3*, genome-wide CRISPR retroviral knock-out library screen identified top-hit genes, *Pparg* and *Bhlhe40*, as important TFs in Th2 differentiation [121]. T helper 17 (Th17) cells are groups of CD4 helper cells, recognized for their indispensable functions in mucosal defences and contributors to inflammatory diseases[142, 143]. Previous studies have identified the combination of critical T cell regulators BATF and IRF4 can remodel chromatin accessibility, initiate Th17 transcription through STAT3, and maintain its characteristics through the lineage-specifying TF, RORγt [142]. Likewise, metabolic reprogramming can affect fate determination of Th17 cells since the lack of amino acids and nucleotides turns T helper cells to Treg lineage[144]. Unbiased in vivo CRISPR screen in primary murine T cells identified Mthfd2 as an essential metabolic checkpoint of Th17 cells, regulating purine biosynthetic and histone methylation. Tregs are crucial cell clusters that mediate immunosuppression and prevent excessive immune activation, significantly contributing to immune homeostasis. Unfortunately, Tregs can strongly inhibit anti-tumour immunity and impair the efficacy of cancer immunotherapies. FOXP3 serves as the representative biomarker of Tregs, while TGF-β and FOXP3 are essential for Tregs differentiation. Mthfd2 deficiency promotes Tregs differentiation due to deficiencies in purine synthesis and decreased nutrient sensor mTORC1 signalling [132]. Genome-wide CRISPR loss-of-function screen identified multiple Foxp3 regulators featured by Brd9-containing ncBAF complex, which promotes Tregs differentiation and hurdle anti-tumour immunity [133]. T follicular helper (Tfh) cells are considered paramount in the initiation and maintenance of germinal centre (GC) and the induction of immunity after infection. An in vivo T cell-intrinsic CRISPR knock-out screen in an acute viral infection setting, integrated with genetic, transcriptomic, and cellular analyses, revealed vital roles of HIF-1α-mTOR and MYC-related pathways in the differentiation of Tfh cells versus Th1 cells [137]. Moreover, recent *in vivo* CRISPR screen and functional validations identified post-translational and metabolic reprogramming factors, namely *ETNK1*, *PCYT2*, and *SELENOI*, could regulate *de novo* synthesis of T cell phosphatidylethanolamine and promote Tfh differentiation by enhancing CXCR5 expression [135]. Though the lineage relationship between Teff and Tmem remains unclear, several TFs and signalling pathways that determine differentiation towards Teff and Tmem have been identified through CRISPR screens. An in vivo CRISPR screen platform targeting CD8^+^ T cells revealed that sgFli1 can alter accessibility at ETS: RUNX binding sites, greatly promoting the conversion to cytotoxic T cells and enhancing Teff-associated gene expression[124]. Additionally, in vivo CRISPR screens in LCMV Arm and chronic Cl13 infection mouse models discovered that Batf and Irf4 cooperate in binding chromatin areas that determine T cell differentiation to Teff [121, 145]. Furthermore, an in vivo CRISPR screen aimed at antigen-specific Tmem cells under Listeria monocytogenes expressing Ova conditions discovered that cBAF and MYC cooperate during T cell division and reprogram the differentiation-related epigenetic landscape, determining T cell fate as terminal effector or memory precursor phenotype [146].

### T cell cytotoxicity

T cell cytotoxicity is characterized by the release of granzymes and perforins and the interactions between death receptors and their ligands. The primary methods to enhance T cell cytotoxicity were to reprogram them to become more tumour-site-located and long-lived, and possess robust effector functions. To address the poor persistence and function of ACTs, an in vivo pooled CRISPR-Cas9 mutagenesis screen revealed that T cells with REGNASE-1 ablation exhibited enhanced functionality and improved persistence in tumour clearance. Additionally, co-deletion of SOCS1 and PTPN2 in *REGNASE-1-null* T cells further enhanced anti-tumour immunity by improving mitochondrial function, indicating a promising therapeutic target for cancer immunotherapies [123, 147]. Genome-wide CRISPR screens in CD4^+^ and CD8^+^ T cells identified that inactivation of SOCS1 could facilitate the release of Th1 effector function cytokines (IFN-γ and IL-2) and improve CD8^+^ T cell cytotoxicity, indicating SOCS1 as a critical intracellular checkpoint in T cell immune responses [97]. To understand the regulators of CD8^+^ T cell infiltration and degranulation in triple-negative breast cancer and glioblastoma (GBM) models, CRISPR screens revealed that RNA helix Dhx37 acted as a negative influencer of granzyme gene expression and IFN-γ production in CD8^+^ T cells [117]. An *in vivo* AAV–Sleeping Beauty-CRISPR screen targeting membrane proteins in CD8^+^ T cells in mouse models of GBM revealed that CAR-T cells with Pdia3 ablation possess enhanced killing ability. In addition, CRISPRi screens identified a group of factors (MALT1, BCL10, TRAF6, and CHUK) that promote IFN-γ secretion in an NF-κB-dependent manner. A CRISPR T cell activation screen concluded that TNFRSF and IL1R1 were intense IFN-γ and IL-2 stimuli [115]. Intriguingly, the output genes of CRISPR screens of T cell cytotoxicity overlapped with genes that govern TCR stimulation and signal transduction. Ablation of negative regulators that hinder TCR response in T cells not only promoted T cell activation but significantly enhanced T cell tumour-killing capabilities through pathways of MAPK (*RASA2*) [131], ubiquitination degradation (*CBLB*) [148], and JAK/STAT (*SOCS1* and *TCEB1*) [149]. CRISPR screens targeting PI3K (phosphoinositide 3-kinase) effectors discovered critical role of RASA3 in T cell migration and functional adhesion mediated by ICAM-1[130]. To overcome the drawbacks that traditional CRISPR loss-of-function screens can merely examine negative regulators of interested functions, novel genome-scale screen strategies were applied in primary human CD4^+^ and CD8^+^ T cells using lentiviral library of barcoded human ORFs to discover positive regulators of T cell functions. When overexpressed in T cells, top hits genes including *LTBR*, *BATF*, and *MAPK3* induce profound transcriptional and epigenomic remodelling, leading to increased T cell effector functions via activating canonical NF-κB pathway [127]. CRISPR screens also shed light on influential factors of Treg functions. A CRISPR pooled screen platform aimed at primary mouse Tregs defined Usp22 and Rnf20 as representative positive and negative Foxp3 modulators [98, 133]. Besides, *HIVEP2* and *SATB1*, which have not been recognized in Tregs function, were identified as hit genes in the network stimulating Tregs-mediated immunosuppression and immune evasion [134]. Nutrients also play decisive roles in determining Tregs function. Insufficient cell metabolism or protein synthesis can hinder signal transduction and Tregs function. A pooled CRISPR screen identified that loss of mTORC1 activator SEC31A and CCDC101 downregulated the expression of amino acid sensor CASTOR1 and impaired Tregs-mediated immunosuppression, resulting in enhanced anti-tumour ability but uncontrolled inflammation [136].

### T cell exhaustion

While immunotherapies have made great strides in various cancers, subsets of patients lack effective responses and develop resistance to T cell-based therapies. Persistent exposure to tumour antigens leads to genetic and functional changes in T cells. In TME, CD8^+^ T cells gradually transform into short-lived effector cells or memory precursor cells to eradicate tumour cells. Continuous stimulation leads to a hyporesponsive status in CD8^+^ TILs characterized by upregulated expression of multiple inhibitory receptors (PD-1, CTLA-4, TIM-3, LAG-3, and TIGIT). Certain TFs including TOX and T-bet govern T cell exhaustion [150]. Numerous studies confirmed that T cell exhaustion is a hallmark of dysfunctional anti-tumour immunity [151, 152]. However, the mechanisms and key factors regulating T cell persistence and exhaustion are still under investigation. A recent genome-wide CRISPR screen based on direct-capture Perturb-seq identified Arid1a and INO80 complex subunits as regulators of CD8^+^ T cell exhaustion [125]. The ablation of these chromatin remodelling factors improved T cell persistence and limited the accessibility of transcriptional and epigenetic hallmarks of exhaustion. Genome-wide gain-of-function CRISPR screens in primary human CD4^+^ and CD8^+^ T cells validated LTBR, a canonically silenced regulator in lymphoid organs, as a synthetic driver of T cell function. When overexpressed in T cells, LTBR induced profound transcriptional and chromatin remodelling changes characterized by upregulation of c-JUN, TCF-1, and BATF3 through triggering canonical NF-κB pathway, leading to enhanced T cell effector function and resistance to exhaustion [127]. Another unbiased in vitro CRISPR screen identified that knocking out RASA2 in T cells could maintain the ability to eliminate tumour cells even with repeated exposure to antigens, which was achieved through elevated expression of genes related to cell cycle, mitochondria, fatty acid metabolism, and enhanced TCR signal transduction [131]. A pooled CRISPR-Cas9 screen revealed that deletion of *SNX9* could inhibit PLCγ1, Ca^2^^+^, and NFATc2-mediated T cell signalling, and reduced the expression of T cell exhaustion-related TFs (NR4A1/3 and TOX), thereby preventing T cell exhaustion and enhancing T cell cytotoxicity [129]. Using in vivo CRISPR screens, regulatory networks controlling T cell exhaustion fate were depicted. IKAROS-deficient cells accumulated as precursor exhausted T cells, which are capable of responding to immunotherapies and contribute to tumour killing[128]. A high-throughput assay for modulators of T cell exhaustion identified several small molecules, among which ingenol mebutate was the most effective one in reversing T cell exhaustion [153]. A genome-wide loss-of-function CRISPR screen focused on post-translational regulation of PD-1 and revealed that the inhibition of Fut8, a core fucosyltransferase, could reduce PD-1 expression through post-translational regulation to prevent T cell exhaustion [120].

### CRISPR screens discover pivotal genes and pathways related to T cell function

**Fig. 6:**
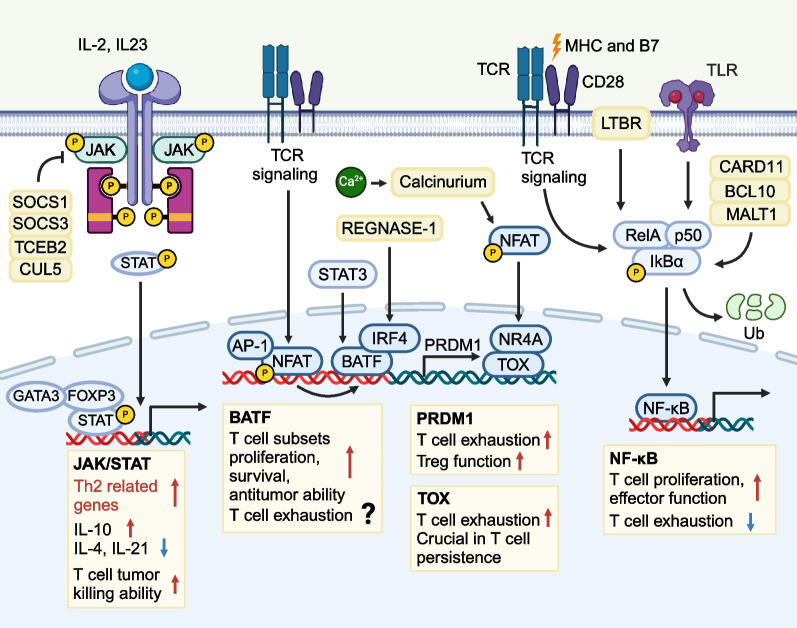
Critical genes and signalling pathways engage in T cell anti-tumour responses. CRISPR screens results have revealed several intriguing genes and pathways that could potentially illuminate ways to enhance T cell functions in cancer immunotherapies. 1) JAK/STAT signalling pathway is essential in various cytokine production and transcription factors generation. Different interleukins and interferons mediate different biological function in T cell effector functions and fate determination. 2) NF-κB signalling pathway is indispensable for TCR signal transduction, and is correlated with enhanced T cell effector function and resistance to exhaustion. 3) BATF is an important AP-1 family transcription factor that functionally cooperates with IRF4 to regulate T cell proliferation, survival, and cytotoxicity. Critical T cell functional transcription factors including T-bet, Runx3, and Blimp-1 are also BATF-dependent. The role of BATF in T cell exhaustion remains unclear. Blimp-1 is highly expressed in exhausted T cells. Blimp-1 deficiency in CD8^+^ T cells indicates a higher possibility of memory cells and lower effector or exhausted phenotypes. TOX is a nuclear factor that maintains CD4^+^ and CD8^+^ T cell development, but leads to exhausted T cell phenotypes. Deletion of TOX in CD8^+^ T cells reverses the exhaustion markers expression, but fails to rescue the impaired effector function against tumours

Genome-wide CRISPR screens are powerful tools to discover promising targets of T cell metabolism and networks of TFs governing effector function. Meanwhile, CRISPR-based pooled screening helps us understand the transcriptional, metabolic, and epigenetic profiles of T cell dysfunction, providing valuable insights into clinical solutions for enhancing T cell responses in cancers. Among the rich screen results, several intriguing genes (*BATF*, *PRDM1*, and *TOX*) and signalling cascades (JAK-STAT and NF-κB pathways) have been identified to extensively engage in multiple branches of T cell anti-tumour responses and might be leveraged to advance cancer immunotherapies (Fig. 6).

### BATF

*BATF* is an important member of AP-1 family TF that consists of a DNA-binding domain and a leucine zipper motif, and was identified as a hit gene in several CRISPR screens [123, 124, 154]. BATF is composed of three members, BATF, BATF2, and BATF3, and negatively regulates AP-1-mediated function by competitively binding with JUN to prevent Jun/Fos heterodimer formation. While BATF2 and BATF3 are mainly expressed in B cells and DCs, BATF is a critical regulator of T cells [155, 156]. Previous studies identified that Batf can cooperate with Irf4 to promote Th17 function by upregulating IL-10 [157], and is involved in the development, differentiation, and effector function of Th2, Th9, Tfh, Treg cells [155, 158–162]. In CD8^+^ T cells, BATF is intimately associated with anti-tumour abilities. An in vivo genome-wide CRISPR screen in REGNASE1-deficient CD8^+^ T cells revealed BATF as a target of REGNASE-1. Deletion of BATF in REGNASE-1-null cells hampered cell proliferation and survival and decreased IFN-γ, GZMB, and TNF-α expression, dampening anti-tumour capability. Moreover, BATF is an essential regulator of CD8^+^ T early differentiation, and BATF-ablated CD8^+^ T cells failed to undergo proliferation or naïve-to-effector transition. Key TFs that govern effector differentiation, proliferation, and survival of CD8^+^ T cells (T-bet, Blimp-1, and Runx3) were highly BATF-dependent [163]. Genome-wide transcriptional profiling further confirmed a regulatory network orchestrated by Batf, Irf4, Runx3, and T-bet, of which Irf family genes were indispensable factors that cooperate with Batf to regulate T cell survival and anti-tumour responses, increasing chromatin accessibility and transcription loci of T cell effector function and persistency under infections [157, 162, 164]. In Tregs, BATF plays a more significant role in regulating the transcriptional networks of tumour-restricted suppressive Tregs. Single-cell transcriptome analysis of CD4^+^ T cells from patients with head and neck squamous cell carcinomas revealed that a subpopulation of Tregs expressing TNFR genes were enriched in TME, and correlated with the poor prognosis of multiple solid tumours. Moreover, CRISPR-Cas9-mediated BATF knock-out in activated Tregs informed that BATF was vital in the stimulation and survival of activated Tregs, suggesting a potential opportunity to therapeutically target tumour-associated Tregs without affecting immune homeostasis [165]. Conflicting results have been presented in the effects of Batf in T cell exhaustion. Some studies discovered that BATF functions as an NFAT–AP-1–driven enhancer when overexpressed in CD19-targeting CAR-T cells. Overexpression of BATF in CD8^+^ CAR-T cells markedly promoted Tmem formation, induced markers of cytotoxicity (GZMB and IFN-γ), and inhibited exhaustion markers (TOX, PD-1, TIM-3, and LAG-3) in TILs [166]. In contrast, an in vitro MSLN-targeting CAR-T cell dysfunctional model found that BATF promoted CAR-T cell exhaustion by binding exhaustion-associated genes, leading to reduced central memory phenotypes and limited cytotoxicity [44]. Similarly, another study concentrated on T cell immunity in active tuberculosis indicated that BATF served as the downstream target of PD-1 signals [167]. Mechanisms underlying BATF in regulating T cell priming, metabolism, proliferation, and effector function require further analysis.

### PRDM1

*PRDM1* encodes BLIMP1, which is identified as a repressor of IFN-β and later proved essential in multiple hematopoietic lineages [168, 169]. BLIMP1 consists of five Krüppel-type zinc fingers at the C terminal and a conservative PR domain at the N terminal, which recruits proteins leading to downstream signalling [170, 171]. BLIMP1 is primarily discovered as a regulator in plasma cells genesis, antibody secretion, embryogenesis, and myeloid lineage development[172, 173]. More importantly, recent studies demonstrated that Blimp1 actively participated in T cell priming, differentiation, effector function, and anti-tumour/viral immunity [174–177]. In CD4^+^ T cells, Blimp1 is the negative influencer of Th1 by competitively combining Th2-related genes (IFN-γ, Tbx21, and Bcl6), leading to Th2 advantageous differentiation. Blimp1 antagonizes Bcl-6 in non-Tfh cells, while high-Bcl-6 and low-Blimp1 expressions are hallmarks of Tfh cells, indicating the repressive role of Blimp1 in Tfh cells. Follicular regulatory T (TFR) cells suppress GC responses via functions of Blimp1 to control IL-23R-CD25 axis and CXCR5-CCR7 axis in TFR cells [178]. Besides, studies assured that Blimp1 was required for Foxp3^+^ Tregs effector function, and Blimp-1 is a target of Foxp3 in Tregs. Deletion of Blimp1 in Tregs repressed the secretion of IL-10 and accelerated the release of pro-inflammatory cytokines (IL-2, IFN-γ, and IL-17), thereby limiting the suppressive ability of effector Tregs [179]. Besides, Blimp1 was demonstrated as a critical regulator governing a population of Tregs located in mucosal sites and secreting IL-10 [180]. In CD8^+^ T cells, Blimp1 regulates the effector function and controls CD8^+^ T differentiation. Blimp1 is also required for T cell cytotoxicity, and the expression of Blimp1 is upregulated when CD8^+^ T cells are exposed to antigens. Interestingly, compared with acute viral infection, chronic viral stimulation induced a more significant upregulation of Blimp1 expression [177, 181]. An ex vivo genetic perturbation using CRISPRi to target PRDM1 in human T cells suggested that BLIMP1 induced memory phenotypes and obviated effector or exhaustion phenotypes [182]. Genetic knock-out of *PRDM1* in CAR-T cells targeting multiple tumour-bearing models facilitated a memory phenotype and prevented T cells from gaining terminal differentiated subtypes, highlighting *PRDM1* as a promising target to improve the efficacy of ACTs [183].

### TOX

TOX is a nuclear factor with heterogeneous expression in different T cell stages, and plays critical roles in T cell life cycle [184]. Though TOX has not been enriched in current CRISPR screenings in T cells, TOX is a crucial downstream element in various signalling pathways associated with T cell differentiation, proliferation, and exhaustion. Several elegant CRISPR screens in T cells identified genes that closely interact with TOX. CRISPR screens have discovered that TOX can promote CD8 + T cell exhaustion in collaboration with NR4A, and TOX serves as a significant downstream regulatory molecule reversing tumour-infiltrating T cell exhaustion with BATF and IRF4, which were the hits in several CRISPR screenings [129]. Moreover, TOX2 is a crucial, constitutively expressed transcription factor in Tfh cells, ensuring the normal functionality of Tfh cells [185].

TOX guarantees T cell maturation, and is highly upregulated during β-selection, and induces positive selection through calcineurin-mediated pathways, whereas the loss of TOX evidently block the CD4^low^CD8^low^ transitional process of positive selection [186–188]. TOX is an important factor that maintains CD4^+^ T lineage development. The transformation of naïve CD4^+^ T cells to Tfh cells is accompanied with TOX upregulation, while *Tox*^*−/−*^ mice experience a decrease in CD4^+^ T cells [189, 190]. Specifically, Tox2 is regulated by Bcl6 and STAT3 in Tfh cells, and Tox2-Bcl6 axis was established as a transcriptional feed-forward loop in Tfh differentiation through promotion of chromatin accessibility [185]. The roles of TOX in T cell exhaustion were increasingly delineated. TOX and NR4A families of nuclear receptors were enriched in tumour-specific dysfunctional T cells and induced by chronic and constitutive stimulation of TCRs and activation of NFAT [191]. Mounting evidence showed that TOX expression in CD8^+^ T cells decreased CD8^+^ T cell infiltration and facilitated T cell exhaustion with elevated PD-1 expression. Moreover, HCC-bearing mice models indicated a correlation between cytoplasm expression of TOX and PD-1, showing that TOX prevented PD-1 from lysosome-mediated degradation, thus accelerating exhausted T cell phenotypes. High levels of TOX in peripheral CD8^+^ T cells indicated poor prognosis in patients with HCC [192]. In human NSCLC and melanoma models, TOX was converged as vital TFs in mediating the exhaustion of TILs through upregulating immune checkpoints in tumour sites [193]. Deletion of TOX in tumour-specific T cells reversed the exhaustion phenotypes with reduced inhibitory receptors and increased expression of TCF-1, but remained a dysfunctional and impaired effector function status. Though TOX was a central regulator of T cell exhaustion, and ablating it prevented differentiation towards exhaustion, TOX prevented overstimulation of T cells and activation-induced cell death, thereby ensuring persistent T cells in tumours [194].

### JAK/STAT signalling pathway

JAK/STAT signalling pathway is a critical cascade that regulates immune responses, transferring rapid signals from membrane to nucleus. JAK/STAT is evolutionarily conserved and contains two main parts, JAK and STAT. JAK family has four members, JAK1, JAK2, JAK3, and TYK2, while STAT family constitutes seven molecules including STAT1, STAT2, STAT3, STAT4, STAT5a, STAT5b, and STAT6. JAK/STAT pathway is activated when activators (GM-CSF, erythropoietin, prolactin, leptin, interleukins, and interferons) bind to cytokine receptors followed by the recruitment of intracellular tyrosine kinases of JAK. Once JAK phosphorylates the tyrosine residues of the receptor, STAT in the cytosol combines to phosphorylated tyrosine residue and undergoes phosphorylation. Phosphorylated STAT dimerizes via SH2 domains, enters the nucleus, and binds to DNA motifs, thus regulating the transcription landscape of multiple processes associated with cancer, inflammation, and autoimmunity. The detailed roles of JAK/STAT family have been reviewed elsewhere[195, 196]. Components of JAK/STAT complexes and JAK/STAT regulators are critical for T cells genesis, survival, and effector function. Genome-wide CRISPR knock-out screen in GBM revealed the indispensable roles of JAK1/2 as IFNγR signalling pathway components in upregulating cell adhesion of CAR-T cells, rendering improved anti-tumour responses [197]. Genome-wide CRISPR-Cas9 screens identified SOCS1, the most potent inhibitor of the JAK/STAT pathway, as a major intracellular negative checkpoint for CAR-T cell activities [97], and TCEB2 complexes, RNF7 and CUL5 to be key suppressors of JAK/STAT signalling in activated T cells [114]. In addition, *JAK1*^*−/−*^ mice were IFN-unresponsive and experienced impaired lymphocytic development due to deficient IL-7 transduction[198]. SOCS3/phosphorylated JAK2/phosphorylated STAT3 could suppress DCs-mediated anti-tumour T cell responses [199], while silencing IL-23R-STAT3 or activating STAT5 could maintain TFR cell stability [178]. Upon cytokine stimulations, distinct STAT family proteins are activated and diversify the routes of CD4^+^ T cell differentiation. STAT1 and STAT4 govern IFN-γ and IL-12 production, respectively, leading to Th1 advantageous polarization. STAT5 and STAT6 induce the downstream IL-4, which further trigger GATA-3 for Th2 differentiation [200–203]. IL-2-STAT5 signalling pathway is essential for TGF-β-mediated induction of Foxp3^+^ Tregs differentiation [204, 205]. STAT3 signalling regulates the expression of Th17-specific genes, IL-17A and IL-17F [205]. Given the versatility of JAK/STAT signalling pathway in T cell function, mutations and abnormal transductions of JAK/STAT signals render individuals susceptible to multiple cancers and immunodeficiency diseases [195, 206–209]. Antibodies against mutant JAK/STAT signal proteins have been developed and proven effective in myeloproliferative neoplasms [206], atopic dermatitis [210], systemic lupus erythematosus [211], and rheumatoid arthritis [212].

### NF-κB signalling pathway

NF-κB signalling pathway is another vital regulator of T cells and has tremendous impacts on reprogramming cytokine production, cell survival, metabolism, and functions. NF-κB signalling pathway responds to various extracellular stresses, antigen exposures, and reactive oxygen species. Dysregulated NF-κB pathway correlates with cancers, autoimmune diseases, and abnormal immune development [213, 214]. The harmonized function of NF-κB signals correlates with not only NF-κB pathway itself, but a vast family of related protein complexes in immune systems, which collaborate and respond to inflammatory signals and are activated through classical and alternative pathways [215, 216]. Regarding T cell signal transduction, NF-κB pathway is the indispensable player of antigen-dependent TCR signalling intranuclear parts [217, 218]. Using single-cell genomics, CRISPR screens identified LTBR as a positive regulator of T cell effector functions and resistance to exhaustion through canonical NF-κB pathway [127]. Genome-wide CRISPR screen in primary human T cells focused on regulators of NF-κB intra-nucleus pathway, and identified positive influencers, MALT1, BCL10, and CARD11, to be key mediators of TCR signalling-dependent T cell proliferation [114]. Coupling CRISPR activation and interference screening further exemplified the involvement of NF-κB pathway key components MALT1, BCL10, TRAF6, and TAK1 in CD4^+^ and CD8^+^ T cells IFN-γ and IL-2 production that enhances type I immune response against intracellular pathogens [115]. More intriguingly, signalosome complex called Carma1-Bcl10-Malt1 (CBM) has critical effects on TCR signalling pathway, leading to NF-κB activation and T cell priming [219]. MALT1 serves as a scaffold protein in CBM complex, recruiting TRAF6 to activate IKK complex. IKK bolsters the proteasomal degradation of NF-κB inhibitor, IκB. Therefore, NF-κB moves from cytoplasm towards nucleus and controls the transcriptions relevant to inflammation responding, T cell activation, and differentiation [220]. MALT1 also promotes T cell activation by cleaving inhibitory NF-κB regulators, A20 and RelB, and facilitates Th17 differentiation via the cleavage of Roquin and Regnase-1 [221]. Moreover, NF-κB pathway regulates T cell subtypes of immune responses in cancers and autoimmunity. Essential functions for NF-κB in modulating Tregs development and function have been revealed [222, 223]. Canonical NF-κB activation proteins including IKK, RelA, and c-Rel ensure the differentiation and maintenance of Tregs and the stable expression of FOXP3 together with post-translational modification [224, 225]. Accordingly, co-deletion of CARMA1 in anti-PD-1 therapy enables the effector activity of Tregs that initiates IFN-γ production and tumour rejection [226]. Studies also discovered Stk4 as an essential regulator of TCR downstream p65-Foxp3-dependent transcription that shapes Tregs-mediated immune tolerance [227]. CARD9 proteins were related to NF-κB integration of different receptors to downstream cellular responses. Patients with homozygous mutations of CARD9 have decreased levels of Th17 cells, which increases the susceptibility to chronic mucocutaneous candidiasis [228].

### Preclinical models to validate CRISPR screen targets

Genome-scale CRISPR screens in T cells have discovered critical influencers that regulate key processes of T cell life cycle. Functional verifications of these highlighted genes and pathways in T cells and CAR-T cells were performed in cell lines and mice models of various cancers. We overviewed these preclinical models in CRISPR screens and hopefully shed light on accelerating the clinical translation of these screen targets of interest.

### In vitro validation

The most common method to identify hits in in vitro CRISPR screens related to effector function is to use primary cells. Primary CD4^+^ and CD8^+^ T cells are extracted from donors, and modified through CAR transduction to create antigen-specific T cells with a specific gene knock-in or knock-out [116], or lentiviral overexpression of target genes [127]. For killing assays of modified T cells, fluorescent protein and luciferase reporter expressing tumour cell lines were introduced [116, 118]. Anti-tumour efficacy and cytotoxic T lymphocyte signature were examined through tracking granzyme B, IFN-γ, IL-2 secretion levels, and flow cytometry analysis of proliferation. Subsets classification of genetically manipulated T cells was conducted to further verify the functional changes and phenotypic alternations [121, 124, 127]. Jurkat cell lines were applied to identify activation-associated regulators due to their immortality and quickly induced activation and TCR transduction characteristics with commonly measured activation markers featured by CD69, phosphorylated ERK, and Rac.

### In vivo validation

T cells are isolated from donor mice and receive transduction with sgRNAs targeting genes of interests. Recipient Cas9 mice are infected with viruses or pathogens to prepare for the infusion of modified T cells. In vivo validation of candidate genes regulating efficacy of GBM immunotherapy uses AAV-*sgMgat5*, *Pdia3*, *Emp1* CD8^+^ TCR transgenic OT-I mice with cognate cOVA model tumour antigen injected, and conducted anti-tumour activity measurement using survival plots, flow cytometry of T cell infiltration, and tumour luciferase imaging [118]. Likewise, the negative roles of Fli1 in CD8^+^ T cells mediating protective immunity against infections and cancers was validated through Fli1-deficiency mice models [124], and SOCS1 as negative checkpoint of CD4^+^ T cells survival and effector function was validated by C57BL/6 mice with Marilyn CD4^+^ T cells [97]. Genetically-modified animals including mice with Cas9 and Tregs-specific ablation of Usp22 and Rnf20 demonstrate previously unknown regulators of Foxp3^+^ Tregs [133]. By co-transduction of sgRegnase-1 lentivirus and control sgRNAs to OT-I cells, and adoptively transferring to B16 Ova melanoma-bearing mice, the significant roles of Regnase-1 and Batf in reshaping effector response of CD8^+^ T cells anti-tumour activity was broadly elucidated, together with the potential role of targeting PTPN2 and SOCS1 in improving therapeutic efficacy of *REGNASE-1* ablated CD8^+^ T cells [123].

### Pharmaceutical targeting and genetic engineering in clinical trials

Small molecules that can directly inhibit target proteins are under investigation through artificial intelligence and computer-aided drug design platform. It was shown that camptothecin, topotecan, and etoposide acted as Fli1 inhibitors, alleviating graft-versus-host disease while resuming the activation and function-related pathways in CD8^+^ T cells [229]. VPC-190444, a small-molecule inhibitor targeting TOX [230], and T-5224, targeting BATF/JUN complex [231], were proved effective to treat T cell-associated lymphomas and protect against mouse osteoarthritis, respectively. More encouragingly, some therapeutics that target the screened genes have entered phase 1/2 clinical trials. The efficacy and safety of NX-1607, a first-in-class oral CBL-B inhibitor, is under investigation in patients with advanced solid tumours (NCT05107674) [232]. Preliminary findings from NCT05643742, a phase 1/2 clinical trial assessing allogeneic CAR-T cells with CRISPR-Cas9-mediated knock-outs of *Regnase-1* and *TGFBR2*, have also demonstrated enhanced CAR-T expansion and improved functional endurance, suggesting potential clinical benefits [233].

## Conclusions

CRISPR screens serve as potent forward genetic tools for investigating mechanisms, phenotypes, and characteristics across diverse applications and species. The CRISPR-based screens of immune cells, particularly T cells, complement and extend previous perturbation methods (RNAi and shRNA), facilitating unbiased discovery of regulators in cellular processes and immune responses.

In vitro CRISPR screens, known for their high efficiency and precise editing with low off-target editing, have successfully elucidated transcriptional and signalling processes in immune and tumour cells. However, their limitation lies in the simplified environment, lacking the microenvironment complexity and cellular interactions present in living organisms. Moreover, in vitro models, consisting of homogeneous populations, overlook the intricate heterogeneity presented in real organisms, potentially leading to inaccuracies in screening readouts. Conducted within living organisms, in vivo CRISPR screens can manipulate genes in a more physiologically relevant environment, thereby better capturing complex cellular interactions and genetic functions in the context of complex biological systems. For in vivo screens, they are more limited in scale compared with in vitro screens. It remains challenging to screen for regulators of T cell priming or naive T cell homeostasis as well as conducting in vivo screens using other types of immune cells [117, 122, 124].

Successful CRISPR screens require the combination efforts in constructing screening models, choosing proper CRISPR methods and CRISPR library, efficient perturbations of cells, proper challenge treatment, and read-outs.

Delivering the CRISPR components into cells with efficiency remains a challenge, which is limited by constraints of the delivery vectors and target cells or organisms, particularly in primary cells and in vivo organisms [96, 116, 118, 119]. Furthermore, it is crucial to overcome the drawbacks derived from CRISPR editing methods featured by the off-target effects, ranging from single base mismatch to consecutive mismatches and nucleotide alternations in CRISPRa/CRISPRi screens, and the limitations of the targeting scope due to restrictive PAM requirements in gain-of-function CRISPR screens [234–237]. For stimuli targeting perturbed cells, it requires deliciated calibration to offer the most physiologically suitable perturbations and improve the physiological relevance between cells and stimuli. New methods for CRISPR-based perturbation such as base editing, prime editing, and high-throughput knock-in are gaining increasing popularity. Equipped with various CRISPR perturbations and the development of high-throughput sequencing platforms, cost-effective, robust, flexible and broadly covering screens will eventually become the primary method of CRISPR screenings [99, 106, 238].

While organisms or tissues in organisms are characterised by heterogeneity and great complexity, CRISPR screenings alone therefore lack richness and multidimensional perspectives when delineating the transcriptional and genomic composition. Therefore, the combination of CRISPR screens with spatial transcriptomics and single-cell sequencing platforms will provide deeper understandings of how gene perturbations in a cell reshape adjacent cells, and the intrinsic patterns of interactions among individual components of the TME and genetic perturbations [99, 106, 239, 240].

T cells are crucially important mediators of cancer immunotherapies, which have demonstrated encouraging clinical responses in a wide range of cancers. Current hurdles of T cell-associated cancer immunotherapies might be mitigated by advanced understandings of molecular circuits that govern T cell biology. High-throughput CRISPR screens have allowed in vitro investigation of critical genes of T cell activation, proliferation, and differentiation [114, 117, 121], and in vivo screening factors modulating TCR-dependent proliferation, T cell cytotoxicity, and fate determination [97, 118, 122, 124]. CRISPR screens combined with pathway mapping and genetic transcriptome profiling could further deepen the understandings of phenotypic and genomic perturbation of critical T cell regulators.

Besides T cells, the efficacy of anti-tumour immunity and cancer immunotherapies is associated with other cellular and noncellular components of TME including tumour cells, NK cells, macrophages, DCs, cytokine expression and enrichment, downstream regulatory networks, and extracellular matrix generated by multiple cells[241–243]. While functional CRISPR screens are widely used in tumour cells[244–246], and DCs[15, 105] to uncover their molecular phenotypes associated with cancer immunotherapies, unbiased integrations of gene functions related to immunotherapies using CRISPR screens are still sparse in most cell types. Genome-wide CRISPR screens in other cells might provide additional insights into cancer immunotherapies.

Though promising results have been achieved in CRISPR screens of T cells and many genetic hits were identified to potentially benefit cancer immunotherapies, it remains a significant knowledge gap to translate those findings into clinical benefits. Very preliminary results suggested that pharmaceutically targeting or genetically modifying these genes hold great promise to potentiate cancer immunotherapies. We look forward to increasing efforts in the selection of small molecules targeting the critical screen targets of T cell functions, with further development of nanoparticle-based drug delivery systems to enable their enrichment in T cells. Collectively, the applications of CRISPR screens to illustrate critical regulators of T cells in mediating anti-tumour responses, together with investigation of targeting these screen hits in clinical practice, will generate promising opportunities in the optimization and application of cancer immunotherapies.

## Data Availability

No datasets were generated or analysed during the current study.

## Abbreviations

ACT: Adoptive cell therapy
ICI: Immune checkpoint inhibitor
CAR: Chimeric antigen receptor
BCMA: B-cell maturation antigen
CRISPR: Clustered regularly interspaced short palindromic repeats
RNAi: RNA interference
ZFN: Zinc-finger nuclease
TALEN: Transcription activator-like effector nuclease
TIL: Tumour-infiltrating lymphocyte
NSCLC: Non-small cell lung cancer
Teff: Effector T cell
Tex: Exhausted T cell
TAA: Tumour-associated antigen
MSLN: Mesothelin
OTOT: On-target, off-tumour
RRMM: Relapsed/refractory multiple myeloma
AML: Acute myeloid lymphoma
ORR: Overall response rate
TME: Tumour microenvironment
CRISPRko: CRISPR knockout
CRISPRa: CRISPR activation
CRISPRi: CRISPR interference
ScCRISPR: Single-cell CRISPR
APC: Antigen-presenting cell
RCC: Renal cell carcinoma
HCC: Hepatocellular carcinoma
DC: Dendritic cell
Tmem: Memory T cell
Treg: Regulatory T cell
DSB: Double-strand break
Cas: CRISPR-associated protein
SgRNA: Single-guide RNA
NHEJ: Non-homologous end joining
TCR: T cell receptor
HLA: Human leukocyte antigen
ORF: Open reading frame
TF: Transcription factor
Th1 cell: T helper 1 cell
Th2 cell: T helper 2 cell
Th17 cell: T helper 17 cell
Tfh cell: T follicular helper cell
GC: Germinal centre
GBM: Glioblastoma
TFR cell: Follicular regulatory T cell
PTP: Protein tyrosine phosphatase
CBM complex: Carma1-Bcl10-Malt1 complex
